# Supplementary material for: Are carbon emissions trading and green financial instruments synergistic? -Comprehensive quantitative research based on content analysis
Source: PLoS One. 2024 Mar 7;19(3):e0298601. doi: 10.1371/journal.pone.0298601 (PMC10919601; doi:10.1371/journal.pone.0298601)
Supplement: S1 Data — (ZIP) [file pone.0298601.s001.zip › Data/地级市Z_F工作报告.zip!/╣≤╓▌/┴∙┼╠╦«╩╨/└┤╫╘╣1⁄2╓┌║┼í╛┬φ┐╦╩2╛▌═°í┐_2018.docx]

各位代表：

现在，我代表市人民政府向大会作工作报告，请予审议，并请市政协委员和其他列席人员提出意见。

一、2017年政府工作回顾

过去一年，在市委的坚强领导下，在市人大、市政协的监督和支持下，我们深入学习贯彻党的十九大精神和习近平总书记在贵州省代表团重要讲话精神，全面落实党中央、国务院和省委、省政府及市委各项决策部署，坚持稳中求进工作总基调，大力推进供给侧结构性改革，着力稳增长、促改革、调结构、惠民生、防风险，全市经济社会发展呈现总体平稳增长、转型逐步加快、质量不断提升、活力明显增强、民生持续改善的良好态势，一些重点领域、重要环节实现新突破、取得新进展，本届政府实现良好开局。

全市地区生产总值完成1461.71亿元，增长11.1%；一般公共预算收入完成138.62亿元，增长6.08%；规模以上工业增加值完成560.27亿元，增长10.2%；固定资产投资完成1652.5亿元，增长21.7%；社会消费品零售总额完成371.94亿元，增长12.1%；金融机构存、贷款余额分别达到1351.29亿元和1071.75亿元，分别增长11.9%和14.6%；城镇、农村居民人均可支配收入分别达到27893元和9069元，分别增长9.5%和10.2%。预计减少农村贫困人口13.5万人，实现3个贫困乡镇“减贫摘帽”，184个贫困村出列，脱贫攻坚再战告捷。

一年来，我们主要抓了以下八个方面的工作。

（一）坚持稳中求进工作总基调，以实干实绩迎接党的十九大胜利召开。强化经济运行调度，着力提高经济发展的质量和效益，规模以上工业企业实现利润总额65.01亿元，增长580%；一般公共预算收入中税收收入完成104.27亿元、增长13.58%，非税收入占比24.78%、下降4.97个百分点，低于全省平均水平。大力培育市场主体，新增3.49万户。启动安全生产强基固本两年攻坚，率先在全省构建“1+N”安全生产监管体系，被列为全国遏制重特大事故试点城市，全市生产安全事故起数和死亡人数实现双降，连续3年未发生重大及以上生产安全事故。成功预报10起地质灾害，紧急转移受胁群众7000多人，全年未发生地质灾害人员伤亡。围绕“迎接十九大·合力保稳定”，建立“3+X”专班机制，安保信访维稳工作系统对接、高效运行、整体推进，实现了“七个坚决防止”目标，人民群众安全感、满意度持续提升。

（二）坚持以脱贫攻坚为统揽，大力推进精准扶贫精准脱贫。深入实施大扶贫战略行动，脱贫攻坚春季攻势、夏季大比武、秋季攻势扎实开展，“四场硬仗”打出成效，预计贫困发生率降至7.3%。围绕打好基础设施硬仗，建成组组通公路3683公里。围绕打好易地扶贫搬迁硬仗，完成搬迁安置10699户42032人，易地扶贫搬迁工作位居全省前列。围绕打好产业扶贫硬仗，实施“五大产业”项目318个，覆盖贫困人口12.2万户31.65万人。围绕打好教育医疗住房“三保障”硬仗，资助农村建档立卡贫困学生2.73万人、资助或减免资金7943.4万元；对贫困人口就医实行“两免除、两提高、三覆盖”，全面落实医疗保障“六个100%”，农村建档立卡贫困人口“四重医疗保障”政策兜底实现全覆盖；大力实施农村危房改造和住房保障三年行动计划，完成农村危房改造4670户，建成小康房2500户。聚焦深度贫困地区，“216”开放式扶贫试验区建设加快。大连市对口帮扶和中国科学院、中国科技大学、鞍钢集团等定点帮扶深入推进，国有企业“百企帮村”、民营企业“千企帮村”等力度加大。

（三）坚持以供给侧结构性改革为主线，加速产业转型升级。认真落实“三去一降一补”，淘汰煤炭落后产能345万吨，商品住房去化周期持续下降，为实体经济企业降低“五大成本”60亿元。大力推进“百企改造”工程，改造企业148户。着力释放先进产能，完成煤炭产量6120.1万吨，采煤机械化程度达到78.3%；完成煤层气抽采量13.75亿立方米，综合利用率达到45.4%，均比上年有所提高。深入实施大数据战略行动，大数据企业主营业务收入预计增长13.6%，智能终端等电子信息产业增加值增长47.5%。玄武岩连续纤维、锰系新材料等新兴产业发展加快。老工业城市转型升级成效明显，得到国务院办公厅通报表扬。大力发展现代农业，粮经比由上年的32：68调整为31：69，畜牧业增加值增长4.7%。水城县现代农业产业园第一批获农业部批准创建国家现代农业产业园，启动创建“1+10”现代高效农业产业园，盘州市、水城县分别成为国家级出口刺梨、茶叶质量安全示范区，猕猴桃、盘县火腿等6个产品获批“生态原产地保护产品”。新增市级龙头企业29家，新增规上农产品加工企业19家，农产品加工转化率预计达到64.1%。大力发展山地旅游业，成功创建3个国家4A级景区，获批3个省级旅游度假区，全年接待游客突破3000万人次，旅游收入突破200亿元，分别增长57%和60%以上。实施服务业市场主体培育工程，新增规上服务业企业58户，贵州首座万达广场在我市建成营业。

（四）坚持生态优先、绿色发展，加强生态建设和环境保护。大力推进生态产业化、产业生态化，绿色经济“四型十五种”产业增加值占GDP比重达到35%。着力守好青山绿水，完成营造林118.4万亩，治理水土流失面积399平方公里、石漠化面积45平方公里，森林覆盖率达到56.94%，“绿色贵州”建设三年行动计划考核排名全省第一。启动实施“以气代煤压减燃煤污染三年行动”，市中心城区年供气能力达到1亿立方米，六枝特区和盘州市均达到5000万立方米，环境空气质量总体保持稳定。建立市县乡村四级河长制，国家“水十条”考核的4个地表水断面全部达到Ⅱ类水质，水城河4个监测断面全部达到功能区水质要求，城镇污水处理率达到87%、比上年提高1个百分点，千人以上集中式饮用水水源地水质监测达标率保持100%。全市首个垃圾焚烧发电项目——六盘水市生活垃圾焚烧发电项目建成投运，小城镇生活垃圾清运实现全覆盖，率先在全省实现县县医疗废物无害化集中处置，城乡生活垃圾无害化处理率达到91%、比上年提高9.94个百分点。落实“五个一批”要求，扎实整改环保问题，中央环保督察组转交的202件案件全部办结。深入开展“六个一律”环保“利剑”执法行动、环保“风暴”专项执法行动，始终保持环保执法高压态势。

（五）坚持统筹城乡发展，加快推进山地特色新型城镇化。新增城镇人口12.09万人，城镇化率提高到49.5%。作为全省第一个地级试点城市，全面启动“多规合一”试点工作。县域乡村建设规划实现全覆盖。启动双水、红桥、董地一体化建设，集中开工项目29个。康乐南路二期工程、麒麟北路、红桥隧道、红桥东路建成通车，钟山、水城、红桥互联互通路网进一步优化。新增污水管网183.8公里、雨水管网102.6公里，集中供热面积达到100万平方米，建成地下综合管廊36.3公里。成功创建国家卫生城市和省级园林城市，“文明在行动·满意在贵州”活动暨“整脏治乱”专项行动综合考核连续4年排名全省第一，在全省率先实现所有县区省级以上文明城市全覆盖，创建全国文明村镇4个、全省文明乡镇25个、全省文明村35个。盘县成功撤县设立盘州市。全面完成小城镇建设“8+X”项目和镇村联动建设任务，水城县玉舍镇入选第二批全国特色小镇。综合整治农村人居环境，开工建设486个农村垃圾处理设施和126个垃圾中转站；打造了20个美丽乡村升级版示范点，20个民族村寨被命名为省级特色民族村寨，六枝特区落别乡牛角村获2017年中国人居环境范例奖。

（六）坚持投资拉动，着力扩大有效投资。实施重大工程和重点项目597个，完成投资1165亿元。启动交通“383”、水利“181”、信息“131”等重大基础设施建设，完成交通投资230.73亿元、水利投资62.4亿元、信息投资12.62亿元。开工建设六赫高速及六枝至黄果树、大坪子至董地、盘州一级环线3条一级公路，安六城际铁路进展顺利，六威高速加快推进，启动实施国省道改造119.9公里、乡镇通二级路184.8公里，村村通油路全面实现。建成2座中型水库，开工建设3座中型水库和9座小型水库，解决了农村9.44万人饮水安全问题。获批国家第三批电信普遍服务试点项目建设城市，窄带物联网实现全覆盖。实施新一轮农村电网改造升级，建成输变电站5个、新增线路1336公里，完成电力投资5.42亿元。积极推进政府与社会资本合作，PPP模式推广工作得到国务院办公厅通报表扬。多渠道加大建设投入，获批省脱贫攻坚投资基金74.77亿元。

（七）坚持改革开放创新，加快释放发展动力活力。深化重点领域和关键环节改革，启动132项改革任务、制定90项改革方案。“三变”改革写入2017年中央1号文件，荣登中国三农十大创新榜，我市被确定为全国农村改革试验区。稳步推进国有企业改革，中央、省属驻市国有企业“三供一业”分离移交有序开展。积极推进机构改革，顺利组建市市场监管局、市生态文明委和市体育局。不断深化“放管服”改革，取消行政许可事项16项、其他行政权力事项8项，下放行政审批事项4项，行政许可事项审查与决定环节的办理期限压缩率达66%。企业“多证合一、一照一码”登记改革全面推进。六盘水海关实现快速通关，成功举办2017中国国际大数据产业博览会六盘水分论坛暨物联网高峰论坛等重大活动，妥乐论坛上升到省级层面举办。引进500强企业4家和一批全国行业龙头企业，全球市场销售额第一的专业索道公司奥地利多贝玛亚进驻六盘水。全市招商引资省外实际到位资金增长8.86%，实际利用外资增长15.6%，外贸进出口总额增长22%。启动建设锰锂电池、玄武岩纤维新材料2个工程技术研究中心，获批筹建国家果蔬检测重点实验室，全市综合科技进步水平指数提高2.19个百分点。民营经济占GDP比重升至57.38%。

（八）坚持以人民为中心的发展思想，持续保障和改善民生。“十件民生实事”年投资计划超额完成。坚持教育优先发展战略，全面推进基础教育综合改革，启动实施基础教育工程三年攻坚，盘州市义务教育基本均衡发展工作通过国家评估认定，水城县“普十五”及“两项督导”工作通过省级督导评估考核验收，农村学前教育营养改善计划实现全覆盖，市二中、市三中成功创建省级二类示范性普通高中，高中教育城建成招生，全市高考一本、本科和专科上线率全省排名均实现进位；六盘水幼儿师范高等专科学校获批筹建；对全市2.9万余名教师实行目标考核，实现教师平均工资收入水平不低于当地公务员，极大调动了广大教师的积极性。在全省率先开展紧密型“医共体”建设试点，深入开展基层医疗卫生服务能力三年提升计划，建成16所“群众满意的乡镇卫生院”和24所甲等中心乡镇卫生院；全市26家县级以上医疗服务机构取消药品加成，实现药品零差率销售。坚守就业民生之本，贫困劳动力全员培训1.45万人，城镇新增就业9.46万人，城镇登记失业率控制在4.2%以内。城市、农村低保标准分别提高10.1%和15.3%，留守儿童、困境儿童、留守老人等特殊困难群体关爱救助进一步加强。城镇保障性住房工作全省考核排名第一。大力实施民族文化产业“10个10工程”，大型史诗舞剧《支格阿鲁》获第六届贵州省少数民族文艺会演剧目金奖、最佳导演奖、最佳音乐创作奖，实现24年来零的突破。凉都国学馆、世界鞭陀文化博物馆等建成开馆。六盘水国际马拉松赛、环中国自行车赛、全国汽车拉力赛、全国水上摩托艇皮划艇大赛、高山滑雪等“一县一品”体育赛事品牌日益成型。与海军部队开展军民共建，“六盘水舰”入列命名。民族、宗教、外事、侨务、对台、人防、档案、妇女、儿童、老龄、残疾人、地方志等各项事业取得新进步。

各位代表！过去一年，我们围绕建设人民满意的政府，全面加强政府自身建设。树牢“四个意识”，坚定“四个自信”，坚持以习近平新时代中国特色社会主义思想为指导，坚决维护以习近平同志为核心的党中央权威和集中统一领导，做到政令畅通、令行禁止。坚持依法行政，出台市政府起草地方性法规草案和制定政府规章程序规定，市县乡法律顾问制度实现全覆盖。推进执法体制改革，完成市县两级综合行政执法机构设置。全面推行“互联网+政务服务”，实现市县乡行政事务“一张网”，打造了全市统一的审批服务平台、公共信用信息平台和应急管理平台。建成全市“双随机、一公开”监管平台。推进权力清单和责任清单融合，涉企经营服务收费目录和县级政府权力清单、责任清单全面公开。认真做好建议提案办理工作，办复率均达到100%。扎实推进政府系统全面从严治党，深入推进“两学一做”学习教育常态化制度化，严格执行中央八项规定和省委、市委十项规定，坚定不移反“四风”、转作风。加强行政监察和审计监督，严肃查处各类腐败案件，政府系统党风廉政建设取得新成效。

在学习实践中，我们深切体会到：对标看齐才能明晰方向，只要向党中央看齐，更加自觉维护习近平总书记的核心地位，牢记嘱托、感恩奋进，就能沿着正确的方向不断前进；依靠人民才能开创未来，只要走好群众路线，增进民生福祉，尊重人民首创精神，始终与群众风雨同舟，就能汇成推动发展的满满正能量；改革创新才能赶超跨越，只要坚持深化改革，激发创新锐气，破除发展障碍，就能释放源源不断的动力活力；厉行法治才能长治久安，只要坚持依法行政，做到法定职责必须为、法无授权不可为，就能取信于民、维护公平；接续奋斗才能行稳致远，只要坚持“一张蓝图绘到底”，发扬“钉钉子”精神，一锤接着一锤敲，一届接着一届干，就能推动经济社会持续健康发展。

各位代表！过去一年取得的成绩，得益于以习近平同志为核心的党中央正确领导，得益于习近平总书记对贵州的亲切关怀，得益于省委、省政府和市委的坚强领导，是市人大、市政协监督支持的结果，是全市各族干部群众团结奋斗的结果。在此，我代表市人民政府，向全市各族人民致以崇高的敬意！向人大代表、政协委员，向各民主党派、工商联、无党派人士、人民团体，向驻市人民解放军、武警官兵和人民警察，向所有关心六盘水建设发展的各界人士，表示衷心的感谢！

在肯定成绩的同时，我们也清醒看到发展中存在的困难和问题。主要体现在：一是脱贫攻坚任务繁重。预计全市还有贫困人口18.6万人，贫困人口依然较多，深度贫困地区交通水利等基础设施较为薄弱，产业扶贫还需加强，越往后脱贫难度越大。二是产业转型升级任务艰巨。传统产业占比依然较大，新的经济增长点支撑作用不强，产业之间、产业内部结构性矛盾突出，结构调整面临不少困难。三是城乡发展不平衡不协调。城乡二元结构、城市二元结构、农村二元结构矛盾依然突出，教育、卫生等公共服务供给不足、质量不高，与人民群众日益增长的美好生活需要还有较大差距。四是防范化解重大风险压力仍然较大。普遍存在融资难融资贵问题，地方政府性债务等风险隐患不容忽视。这些问题，是发展质量不够高的具体体现，也是发展不平衡不充分的短板所在，必须采取有效措施加以解决。

二、2018年政府工作主要任务

2018年，是全面贯彻党的十九大精神的开局之年，是改革开放40周年，是决战脱贫攻坚、决胜同步小康、实施“十三五”规划至关重要的一年，也是六盘水建市40周年，做好2018年工作事关全局、意义重大。我们要按照中央和省的决策部署，拥抱新时代、聚焦新目标、落实新部署、实现新作为，为到2020年与全国全省同步全面建成小康社会奠定更加坚实的基础。

今年政府工作的总体要求是：全面贯彻党的十九大精神和习近平总书记在贵州省代表团重要讲话精神，以习近平新时代中国特色社会主义思想为指导，坚持稳中求进工作总基调，坚持新发展理念，紧扣社会主要矛盾变化，按照高质量发展的要求，统筹推进“五位一体”总体布局和协调推进“四个全面”战略布局，大力培育和弘扬新时代贵州精神，坚持以供给侧结构性改革为主线，统筹推进稳增长、促改革、调结构、惠民生、防风险各项工作，大力推动质量变革、效率变革、动力变革，深化改革开放，保障改善民生，全力打好防范化解重大风险、精准脱贫、污染防治“三大攻坚战”，全力守好发展、生态、安全“三条底线”，全力推进大扶贫、大数据、大生态“三大战略行动”，创新发展思路，发挥后发优势，决战脱贫攻坚，决胜同步小康，全面建设统筹城乡改革和发展先行市，奋力开启新时代六盘水经济高质量发展新征程，奋力续写新时代六盘水改革发展新篇章。

全市经济社会发展的主要预期目标是：地区生产总值增长12%左右，一般公共预算收入增长6%，社会消费品零售总额增长13%，固定资产投资增长20%左右，金融机构存、贷款余额分别增长18%和22%，城镇、农村居民人均可支配收入分别增长9%左右和10%左右，城镇登记失业率控制在4.2%以内，森林覆盖率达到59%以上，完成省下达的节能减排降碳任务。

围绕实现全年目标，重点抓好以下十个方面工作。

（一）全力打好防范化解重大风险攻坚战。时刻绷紧防范化解重大风险这根弦，建立完善风险防范和处置机制，确保各个领域、各种类型风险有人管、有人防、有对策。强化政府性债务风险管控，力争今年6月底前完成存量政府性债务置换，有序推进展期降息和债务剥离，多渠道筹集资金偿还到期债务。严格落实政府性债务管理“七严禁”，坚决查处违法违规举债，防止形成新的隐形债务。推进投融资平台公司市场化实体化转型，进一步理顺管理体制机制，改进监管方式，提高监管效率。用好收费公路专项债券、土地储备专项债券、PPP模式等方面的政策措施，用好省脱贫攻坚、交通产业、文化产业等省级基金和凉都“三变”扶贫产业基金，依法依规拓宽融资渠道。强化金融领域风险管控，建立健全监测预警和早期干预机制，加大融资性担保机构、小额贷款公司、权益类交易场所的日常业务监管力度，持续做好防范、打击和处置非法集资工作，守住不发生系统性金融风险的底线。强化政府投资项目风险管控，加强项目前期工作，严格执行基本建设程序，加大过程监督管理力度，从严控制工程造价，切实保证工程质量。强化安全生产和社会矛盾风险管控，深入开展安全生产大检查和地质灾害隐患大排查，加强防灾减灾救灾能力建设，推进遏制重特大事故试点城市建设，完成安全生产强基固本两年攻坚任务和国家安全生产领域改革发展联系点工作年度任务，有效防止较大以上生产安全事故发生。深化“平安凉都”建设，实施“雪亮工程”，创新完善预防化解社会矛盾机制和社会治安防控体系，严厉打击各类违法犯罪活动。

（二）全力打好精准脱贫攻坚战。深入实施大扶贫战略行动，启动实施农村贫困家庭收入三年倍增行动计划，按照“两翼先行、中路突破，全面决胜，确保如期全面小康”的总体思路，聚焦深度贫困地区，更加突出重点、更加注重质量、更加压实责任，扎实开展“脱贫攻坚作风建设年”行动和“领导干部轮战深度贫困村”行动，高质量打好“四场硬仗”，巩固盘州、决战六枝、攻坚水城，扎实做好国家对盘州市贫困县退出的评估检查迎检工作，全力推进六枝特区“减贫摘帽”，减少农村贫困人口10万人，213个贫困村出列。继续打好基础设施硬仗，认真贯彻落实习近平总书记关于“四好农村路”的重要指示要求，加快“组组通”公路项目建设，深度贫困地区全面实现组组通硬化路。继续打好易地扶贫搬迁硬仗，全面建成2017年安置点项目，确保今年6月底全部搬迁入住；全面完成2018年安置点项目主体工程；完善“五个三”生计保障和后续发展配套措施，户均就业率、搬迁群众子女入学率、公共服务覆盖率均达到100%，加快把安置点建成和谐社区、活力社区。继续打好产业扶贫硬仗，规模化发展“五大产业”，分别完成蔬菜、食用菌、茶叶、中药材种植面积88万亩、0.3万亩、50万亩、10万亩，生态家禽出栏1400万羽；巩固传统烤烟特色产业，完成种植12万亩；争取更多省脱贫攻坚投资基金，在四个县市区打造“一县一业”全产业链项目，实现贫困群众产业扶贫全覆盖。继续打好教育医疗住房“三保障”硬仗，全面落实教育资助政策，确保义务教育阶段贫困学生入学率达到100%；对贫困劳动力进行全员培训，确保每户有劳动能力的贫困家庭至少1人熟练掌握一门实用技能；强化“四重医疗”保障，完成五级远程医疗体系建设，提高群众就近就医水平，农村建档立卡贫困人口家庭医生签约服务率达到100%；大力推进“六个攻坚”，严格落实“危改”工程“五主体，四到场”制度，实现建档立卡贫困户等四类重点对象危房改造全覆盖。落实好与大连市签署的东西部扶贫协作助推脱贫攻坚合作协议，项目化推进定点帮扶和社会帮扶。实施生态扶贫，新增建档立卡贫困人口转化为生态护林员1450名。始终把精准贯穿脱贫攻坚全过程，围绕“三率一度”，对贫困户纳入、退出情况及致贫原因、脱贫措施等进行逐户核实，强化动态管理，实行差别化扶持、精准化帮扶。坚持扶贫与扶志、扶智相结合，激发和培育贫困群众脱贫致富的内生动力。

（三）全力打好污染防治攻坚战。深入实施大生态战略行动，下更大决心整治环境问题，下更大力气推进生态建设，促进生态环境质量总体改善。认真落实中央环保督察组督察反馈意见整改方案，实施污染防治攻坚行动，持续推进“双十”工程，有效解决重点领域、重点行业、重点企业治污减排问题。加大大气污染治理力度，推进“以气代煤压减燃煤污染三年行动”，严控扬尘污染，坚决打赢蓝天保卫战，市中心城区环境空气质量优良率和主要污染物PM10、PM2.5浓度控制在省下达目标任务内。持续改善水环境质量，严格落实河长制，推行湖长制，推进重点流域污染治理，加快饮用水水源保护区规范化建设，开展城镇污水配套管网建设攻坚，千人以上集中式饮用水水源地水质达标率保持100%，省控以上断面地表水水质优良率达到94%以上。加强土环境治理，开展废渣及危险废物处理设施建设攻坚，引导建材企业规模化利用工业固体废弃物，煤矸石综合利用率达到60%以上，危险废物得到依法安全处置。大力推进新一轮绿色贵州建设行动计划，完成营造林60万亩以上，治理水土流失面积170平方公里、石漠化面积60平方公里。实施绿色经济倍增计划，开展绿色制造三年专项行动，大力发展绿色经济“四型十五种”产业，创建绿色示范企业2家以上，绿色经济占GDP比重力争达到40%。加强矿山生态保护和恢复，加大地质灾害防治力度。推行生态环境损害赔偿制度，加大环境执法监管力度，建立“查、处、移”一条线和快查快办快移的行政处罚模式，探索构建“1+N”环境保护管理体系。

（四）大力推进工业转型升级。按照“立足煤、做足煤、不唯煤”的发展思路，深化工业供给侧结构性改革，持续推进“三去一降一补”，积极申建国家老工业城市转型升级示范区，加快国家循环经济示范城市创建，推动企业高端化、绿色化、集约化发展，规模以上工业增加值增长10.6%。继续以“双百工程”为抓手，以实体经济为主攻方向，积极引进优强企业参与我市“百企改造”，改造企业100家以上。大力实施市场主体培育“四转”工程，新增入库规上工业企业75户。坚持把煤炭产业作为战略性支柱产业，狠抓煤电产业转型升级，进一步提升煤炭工业发展质量，启动20处煤矿智能机械化改造，采煤机械化率达到80%以上，淘汰落后产能93万吨以上。加快四个煤电一体化基地建设，加快兼并重组批复的大中型矿井建设，建成化乐煤矿。加大煤层气抽采利用力度，抽采量达到15亿立方米、综合利用率达到46%。建立健全能源工业运行新机制，抓好电煤保供，完成发电量380亿千瓦时以上。加快煤炭交易市场体系建设，建立发布煤炭价格指数体系，形成煤炭交易价格新机制。推动“煤电+下游产业”发展，延长煤焦化及下游加工产业链，加快煤化工项目技改升级，建成铝王铝业、十九度铝业等深加工项目，形成电解铝全部就地深加工的生产能力。支持首钢水钢集团“绿色工厂、智能工厂”建设。大力发展新材料、新能源、电子信息、现代装备制造等新兴产业，建成投产玄武岩二期项目，启动建设10万吨高纯硫酸锰项目二期生产线和万吨级氢氧化锂项目。以建设多贝玛亚索道公司贵州工厂为契机，培育壮大旅游装备制造业。按照工业进区、产业入园、污染集中治理的要求，优化园区要素配置，高效盘活标准厂房，加快完善园区基础设施、环保设施、服务设施，推动产城融合、产业成链、企业发展，在5个省级开发区分别形成2个全产业链企业集群，在其他园区分别形成1个以上全产业链企业集群，园区产值占规上工业比重达到40%以上。开展质量提升行动和对标达标专项行动。大力培育现代产业工人，提高劳动力供给的数量和质量，建设知识型、技能型、创新型劳动者队伍。大力推动创新驱动发展，改善创新环境，申报高新技术企业20家、培育科技成长梯队企业5家以上，大力推进国家级高新技术产业园区创建工作，加强科教合作和人才引进培育，全市综合科技进步水平指数达到54%。

深入实施大数据战略行动，加强顶层设计，加快推进“互联网＋”行动，整合数据资源，建好数据平台，消除信息孤岛和数据烟囱，全力打造“智慧凉都”。推动大数据与实体经济深度融合。对有条件的规上工业企业实施大数据、物联网、智能制造等技术改造，培育大数据龙头企业4家以上。加快物联网建设和运用，推动窄带物联网与“智慧凉都”建设融合发展，建成智慧城市体验中心，积极实施省级智能制造试点示范项目。大力发展智慧农业，在“1+10”现代农业产业园全面推广物联网技术运用，建设2个农业园区物联网试点示范。大力发展智慧旅游，加快国家4A级景区智能化升级，建成市级智慧旅游平台。建设六盘水市工程车协同管理大数据云平台，实现建筑行业智能物流及绿色生产全面数据化监管。实施电子商务“百乡千村”工程，支持和鼓励年轻人开办电商。推进政府数据“聚通用”，完成已迁入“云上贵州”应用系统100%数据目录梳理、100%绿色数据上架，实现跨行业、跨部门数据共享，加快建设政府大数据应用典型示范项目。推动遥感技术在地质灾害、安全生产等领域的运用，提高技防能力和水平。继续办好中国国际大数据产业博览会六盘水分论坛。

（五）大力实施乡村振兴战略。坚持农业农村优先发展，按照产业兴旺、生态宜居、乡风文明、治理有效、生活富裕的总要求，推进农业农村现代化，让农业成为有奔头的产业，让农民成为有吸引力的职业，让农村成为安居乐业的美丽家园。坚持以农业供给侧结构性改革为主线，狠抓特色农业转型升级，加快建设现代农业，着力提高农业发展质量和效益，一产增加值增长6.8%。围绕构建现代农业产业体系，坚决落实“旱地基本农田全部种植经济作物”的要求，调减玉米种植面积142.27万亩，巩固提升“3155工程”，确保投产面积达到150万亩以上，经济作物占种植业的比重进一步提高；实施草食生态畜牧业裂变发展计划，畜牧业增加值占比提高1个百分点以上；以农产品加工为重点促进一二三产融合发展，突出抓好刺梨、小黄姜、猕猴桃加工，以及肉类加工等项目，争取岩博酒业在“新三板”挂牌，“盘县火腿”生产规模达到6000吨，新增规上农产品加工企业20家。围绕构建现代农业生产体系，把提高农业技术水平放在质量兴农的重要位置，启动“科技小院”建设试点，大力推动农业科技创新和成果应用，积极开展农业新技术新品种研究及试验示范，因地制宜推广良种良法。大力发展设施农业，打造1—2个具有特色的农业科技园区；支持“1+10”现代农业产业园建设，新增省级园区、省级引领型园区各1个，力争水城县国家现代农业产业园通过国家评估，支持盘州市申报创建国家现代农业产业园。强化质量兴农、品牌强农，推进农业标准化生产、全程化监管，加快生态原产地产品保护示范城市建设，推进六盘水全域生态产品质量可追溯体系建设。围绕构建现代农业经营体系，大力培育新型职业农民，鼓励和引导大学毕业生、返乡农民工回乡发展现代农业；大力培育新型经营主体，新增省级龙头企业41家、市级龙头企业30家。推动伊赛、大北农、温氏集团、粤旺集团等种养殖项目加速落地、早日建成，充分发挥企业在建设基地、做大产业、开拓市场、带动增收等方面的作用；建立完善利益联结机制，全面推广“龙头企业+合作社+农户”模式，细化农户在产业链、利益链中的环节和份额，让小农户通过多种途径和方式进入规模经营、现代生产，分享农业现代化成果，实现持续稳定增收。

推进新一轮“四在农家·美丽乡村”小康行动计划，打造美丽乡村升级版。坚持规划先行，深入开展村庄规划大会战，实现行政村和30户以上居民点村庄规划全覆盖。走乡村绿色发展之路，开展500个行政村“三型”村庄建设，全面整治农村人居环境。积极推进农村“厕所革命”，大力开展农村户用卫生厕所建设和改造。坚持农村基础设施建管并重，鼓励农民投工投劳参与建设和管护，坚决杜绝“只建不管”造成资源闲置和浪费。健全自治、法治、德治相结合的乡村治理体系，推进市县两级文明村镇创建工作，开展移风易俗行动，培育文明乡风、良好家风、淳朴民风，依托村规民约、教育惩戒等褒扬善行义举、贬斥失德失范，唱响主旋律，培育新风尚。

（六）加快发展现代服务业。狠抓特色旅游业转型升级，推动旅游业从高速增长转向优质发展，全年接待游客、旅游总收入分别增长40%和50%以上。高质量编制完成全市全域旅游发展总体规划和北盘江流域旅游开发建设“三规合一”规划。加大旅游资源开发统筹力度，除需省级审核的旅游开发项目外，旅游资源大普查发现的三级及以下旅游资源的开发利用或投资规模为2—3亿元的旅游项目由市级审核，把重点放在提升现有景区景点的建设、管理和服务水平上。以承办第十五届贵州旅游产业发展大会为契机，开展精品旅游景区创建活动，完善提升旅游公路、游客集散中心、旅游厕所等配套服务设施，新增国家4A级旅游景区和省级旅游度假区各2家以上，启动国家级旅游度假区和国家5A级景区创建工作。引进实力强、业绩好的运营商参与现有景区景点经营，提高市场化专业化运营水平，新增4家以上品牌管理企业参与景区运营、8家以上品牌管理企业参与酒店经营。进一步激发旅游公司、旅行中介机构等营销积极性，充分利用互联网手段，有力有效有针对性地做好旅游宣传推介工作。大力发展现代商贸、现代物流、研发设计等生产性服务业，加快建设便民商业设施，新增省级服务业集聚区1个以上。加快构建城乡一体化智能化物流运输配送体系，新增冷库库容5万吨、冷链物流专车50台，实现县域冷库全覆盖。积极发展航空货运包机业务。实施新一轮大健康产业行动计划，大健康产业增加值增长15%以上。加快发展居民和家庭服务、法律服务等生活性服务业，支持社会力量发展养老服务业。

（七）持续扩大有效投资。充分发挥项目建设对投资的拉动作用，优化投资结构，提高投资的质量和效益。加强项目库建设，强化项目动态管理和“六个一”项目服务。着力提高工业投资比重，完成工业投资536亿元。大力推进以交通“383”、水利“181”和信息“131”工程为重点的基础设施建设，分别完成交通、水利和信息投资267亿元、75亿元和15亿元。基本建成安六城际铁路并具备联调联试条件，开工盘州至兴义城际铁路，加快推进水盘城际铁路前期工作。建成六威高速公路，加快普盘高速、纳兴高速前期工作并力争开工建设，加快玉普高速公路前期工作并争取纳入省高速公路网加密规划，推进六赫高速及大坪子至董地、发箐至金盆、六枝至黄果树等一级公路建设，完成国省道改造60公里。开工建设月照机场改扩建工程、盘州官山机场试验段。加快推进英武水库前期工作，开工建设2座中型水库、10座小型水库。深入实施光网城市、“宽带乡村”等工程，建成全光网市，行政村光纤网络、4G网络全覆盖。推进新一轮农村电网改造，新增输配电线路765公里。落实国家激发民间投资政策措施，推动政金企对接，鼓励和支持符合条件的企业上市融资，积极拓宽投融资渠道。

（八）加强城镇规划建设管理。坚持以人的城镇化为核心，围绕建设山地特色现代化城市，推进双水、红桥、董地一体化建设，做大做强市中心城区，培育壮大盘州市和六枝特区城区，全面放宽各类转移人口落户限制，推行居住证制度，全市常住人口城镇化率提高2个百分点。用好“多规合一”试点成果，加快城市开发边界划定，实现空间开发全程监管。推动城市扩容提质，做好德坞、官寨、火车站、塔山等棚户区城市设计和改造，启动鱼塘片区等规划建设。加快完善城市交通路网，加快乌蒙大道、凤凰路东段、人民路东段建设，开工建设钢城大道二期、六威高速连接线与麒麟北路立交互通，提升改造水西北路和人民路水西路至麒麟路段，缓解城市拥堵，积极推动解决城市停车难问题。加快推进集中供热项目，全面完成地下综合管廊试点建设任务并投入试运营。规范提升物业管理服务水平，优化社区生活服务设施布局，打造城市社区一刻钟服务圈，培育和构建守望相助的社区文化。加快10个示范小城镇建设，启动建设10个特色小镇，郎岱和玉舍两个国家特色小镇打造取得明显成效。继续推进“创文”工作。进一步提升园林城市建设水平，启动市中心城区“城市双修”，推进森林城市和海绵城市建设，大力发展绿色建筑，提升宜居水平。

（九）加大改革开放力度。坚持改革推动、开放带动、创新驱动，强化高质量发展的动力支撑。围绕全国农村改革试验区建设，推进承包地“三权”分置，加大财政资金整合使用和金融保险支持力度，建成市县乡村四级联动的农村产权交易平台，巩固农村“三变”改革成效，着力打造“三变”改革升级版，规范提升“三变”改革示范点59个、示范乡镇12个、股权示范村17个。持续深化行政体制和“放管服”改革，开展“减证便民”行动，大力压缩审批流程，提升政府服务水平，力争政务服务大厅事项办理“最多跑一次”达到80%以上。围绕激发市场活力，推进商事制度改革，全面实行市场准入负面清单管理、“多证合一”、简易注销登记，推进“证照分离”。支持民营经济加快发展，民营经济占比提高到59%。积极推进国有企业改革和重组，妥善处置困难职工安置、企业产权和债务纠纷等问题，继续抓好中央、省属驻市国有企业“三供一业”分离移交工作，分别组建市商贸物流和供销企业。稳步开展农业水价综合改革、殡葬服务改革，统筹推进生态文明体制、盐业监管体制、财税体制、司法体制、人事制度等改革。

实施开放带动战略，积极融入“一带一路”和长江经济带建设，深化与贵阳、大连等地在经贸、科教等领域的交流合作。用好省数博会、酒博会、茶博会等平台，把我市优质产品推出去，把优强企业、高端人才、先进技术引进来。继续办好妥乐论坛，加强与东南亚国家的经贸往来。充分发挥六盘水海关辐射带动作用，积极申建六盘水保税物流中心和红果公用保税仓库，引导和支持现有外贸企业发展成为进出口主体，全年完成外贸进出口总额3.8亿美元，实际利用外资2.5亿美元。构建“亲”“清”新型政商关系，优化营商环境，进一步激发市场活力和大众创业、万众创新的潜力。开展“产业大招商突破年”行动，突出全产业链招商，推广第三方招商，引进省外到位资金950亿元，引进500强企业4家以上，落地省外投资项目500个以上。

（十）大力保障和改善民生。牢固树立以人民为中心的发展思想，把高质量发展的落脚点放在提高保障和改善民生水平上、放在满足人民群众多层次多样化的美好生活需要上。坚持教育优先发展，大力实施基础教育工程三年攻坚，完成“全面改薄”计划，启动397所各级各类学校新建改扩建项目。加快义务教育基本均衡发展，六枝特区、水城县、钟山区通过国家评估认定。推动高等教育内涵式发展，六盘水幼儿师范高等专科学校实现招生。加快发展职业教育、特殊教育，支持民办教育健康发展，加强青少年心理健康教育及社区教育。扎实推进基础教育综合改革，开展中小学校长职级制改革等试点。坚持就业优先战略和积极就业政策，完善就业创业联动协调工作机制，打造市中心城区“一站式”创业服务指导站，发放创业担保贷款3.2亿元以上，实现5000名以上大学毕业生和5.6万名以上农民工就业创业，新增城镇就业9万人以上。推进健康六盘水建设，加快“百院大战”项目建设，启动实施新一轮改善医疗服务三年行动计划，重点推进医疗卫生“五个全覆盖”；全面建成市县乡三级紧急医学救援中心，50%的乡镇建成中医馆；巩固国家卫生城市创建成果，全市所有乡镇创建省级以上卫生乡镇；加快公立医院综合改革，全面推开“医共体”改革，加快构建县乡村联动的县域医疗服务体系；扎实做好计划生育服务管理。严格落实食品药品安全责任制，加快创建国家食品安全示范城市。深入实施“全民参保计划”，推进工伤保险“同舟计划”，推行安全生产责任保险，社会保险覆盖率达到94%以上；推进单病种付费模式改革，提高异地就医直接结算覆盖面。稳步提高城市和农村低保标准、优抚补助标准、城乡居民基础养老金最低标准，做好特困人员、残疾人兜底保障，加强对未成年人、困境儿童、失独失能老人和农村“三留守”人员关爱保护，加强城镇困难职工解困脱困工作。大力发展文化体育事业，深度挖掘和合理开发“三线”文化、民族文化、历史文化和红色文化，加强文化遗产和自然遗产保护，开展六枝地宗矿等3个文化产业示范点建设，市档案馆、市图书馆、市文化馆、市地方志馆、市科技馆新馆投入使用，积极申报创建全国第四批公共文化服务体系示范区，推进海坪彝族文化核心区申报省级文化园区；加强基层公共文化服务，广泛开展群众性文化活动，继续办好消夏文化节。统筹群众体育、竞技体育、体育产业发展，广泛开展全民健身，积极探索采用市场运作手段提升六盘水夏季国际马拉松赛、国际滑翔伞赛等品牌赛事举办水平；加快老王山国家生态型多梯度户外运动基地、野玉海户外运动基地建设，启动牂牁江生态体育公园申报工作。支持军队、武警部队建设和监察委、法院、检察院工作。进一步加强国家安全、宗教、外事、侨务、对台、人防、档案、老龄、残疾人、地方志等工作。

扎实办好“十件民生实事”。（1）基本解决中心城区义务教育阶段66人以上“超大班额”问题；（2）新建、改建公办幼儿园80所；（3）实施城镇低收入困难群体脱困帮扶教育资助工作；（4）建成农村通组公路3868公里和实施农村公路安全生命防护工程；（5）实现深度贫困村和易地扶贫搬迁集中安置点卫生室规范化建设全覆盖；（6）实施贫困劳动力全员培训工程，完成22347人培训任务；（7）解决建档立卡贫困人口2万人安全饮水问题；（8）改造农村危房9005户；（9）继续实施市中心城区集中供暖项目，新增集中供热面积180万平方米；（10）新增多彩贵州“广电云”户户用工程用户30万户。

三、加强政府自身建设

新时代对政府工作提出了新的更高要求。我们将围绕做到信念过硬、政治过硬、责任过硬、能力过硬、作风过硬，大兴调查研究之风、真抓实干之风、勤俭节约之风，践行政策设计、工作部署、干部培训、监督检查、追责问责“五步工作法”，不断提高施政能力和服务水平，努力建设人民满意的服务型政府。

（一）强化政治意识，建设忠诚可靠的政府。坚持“三个一以贯之”，树牢“四个意识”，坚定“四个自信”，严守政治纪律和政治规矩，始终把政治建设摆在首位，坚决维护习近平总书记在党中央和全党的核心地位，坚决维护党中央权威和集中统一领导。深入推进“两学一做”学习教育常态化制度化，扎实开展“不忘初心、牢记使命”主题教育，自觉用习近平新时代中国特色社会主义思想武装头脑、指导实践、推动工作；自觉把忠诚和看齐写在岗位上、融入事业中，做到中央有号召、省委有要求、市委有部署，市政府就有行动、有落实。

（二）强化法治意识，建设依法履职的政府。坚决维护宪法权威，依宪施政、依法行政，严格依照宪法法律行使职权、履行职责、开展工作。强化科学民主决策，完善重大行政决策程序制度、合法性审查制度。严格规范公正文明执法，推行行政执法公示、执法全过程记录、重大执法决定法制审核制度。自觉接受人大及其常委会的法律监督、工作监督，自觉接受政协的民主监督，认真办理人大代表建议和政协委员提案，高度重视民主党派、工商联和无党派人士的意见建议。加强政务公开，主动接受社会和舆论监督。

（三）强化民生意识，建设公心为民的政府。坚持执政为民，在发展中保障和改善民生，既尽力而为、又量力而行，一件一件抓紧抓实、抓出成效。增强群众观念和群众感情，坚持民生导向和问题导向，面对面、心贴心、实打实地做好群众工作，用贴近实际和贴近群众的务实举措，着力解决群众反映强烈的突出问题，千方百计解民忧、纾民困、惠民生。大力推动社会治理重心下移、人员下沉，最大程度便民利民，不断提高人民群众满意度。

（四）强化创新意识，建设开拓进取的政府。以勇于自我革命的精神，进一步解放思想，大胆开拓创新，只要符合中央和省精神，只要符合六盘水实际和群众意愿，就大胆试、放手干、坚决改。大力建设学习型政府，增强创新本领，积极运用互联网技术和信息化手段开展工作，拓展提升政府治理能力。在全面落实中央和省各项决策部署的前提下，沿着中央指明的方向，结合六盘水实际，努力打造可复制可推广的改革创新试点示范。

（五）强化责任意识，建设担当作为的政府。以“明责”立规矩，健全一一对应的责任链、任务链，明确完成时限和责任人，确保每项工作有人抓、有人管、有落实。强化担当精神，把高标准履职尽责作为基本要求，做到日常工作能尽责、难题面前敢负责、出现过失敢担责。强化知行合一，把狠抓落实作为履职尽责的检验标准，做到提高政治站位抓落实、转变作风抓落实、提高能力抓落实、讲究方法抓落实。强化督查问效，既督任务、督进度、督成效，又察认识、察责任、察作风，抓住不落实的事、问责不落实的人。坚持严管和厚爱结合、激励和约束并重，健全干部激励评价和容错纠错机制，为担当者担当、为干事者撑腰。

（六）强化效益意识，建设高效务实的政府。牢牢把握高质量发展这一根本要求，坚持质量第一、效益优先，提高全要素生产率，促进经济结构优化升级。更加突出市场配置资源的决定性作用，更好发挥政府作用，优化资源配置，让投资有回报、产品有市场、企业有利润、员工有收入、政府有税收、环境有改善。牢固树立过“紧日子”的思想，严格预算执行管理，强化刚性约束，规范经费审批，切实把有限的资金用在“刀刃”上。发扬求真务实、真抓实干的作风，既增强紧迫感，又克服一蹴而就的思想，不搞“花架子”、不做表面文章，推动干部作风“脱虚向实”，以苦干实干提高发展效益。

（七）强化廉政意识，建设清正廉洁的政府。自觉扛起全面从严治党主体责任，严格落实“一准则、两条例”，始终保持政府系统反腐倡廉高压态势。严格遵守中央八项规定及实施细则、省委十项规定及实施细则、市委十项规定及实施意见，驰而不息反“四风”。进一步完善权力运行监督机制，实现对所有行使公权力的公职人员监察全覆盖、公共资金使用审计全覆盖，推动公共资源交易全过程公开，做到干部清正、政府清廉、政治清明。

各位代表！团结凝聚力量，奋斗成就伟业。让我们紧密团结在以习近平同志为核心的党中央周围，在市委的坚强领导下，不忘初心、牢记使命，不负嘱托、感恩奋进，以时不我待、只争朝夕的精神投入工作，奋力续写新时代六盘水改革发展新篇章，向党和人民交上满意的答卷！
